# U12 intron positions are more strongly conserved between animals and plants than U2 intron positions

**DOI:** 10.1186/1745-6150-3-19

**Published:** 2008-05-14

**Authors:** Malay Kumar Basu, Wojciech Makalowski, Igor B Rogozin, Eugene V Koonin

**Affiliations:** 1 National Center for Biotechnology Information, National Library of Medicine, National Institutes of Health, Bethesda MD, USA; 2 Institute of Bioinformatics, Faculty of Medicine, University of Muenster, Germany

## Abstract

We report that the positions of minor, U12 introns are conserved in orthologous genes from human and *Arabidopsis *to an even greater extent than the positions of the major, U2 introns. The U12 introns, especially, conserved ones are concentrated in 5'-portions of plant and animal genes, where the U12 to U2 conversions occurs preferentially in the 3'-portions of genes. These results are compatible with the hypothesis that the high level of conservation of U12 intron positions and their persistence in genomes despite the unidirectional U12 to U2 conversion are explained by the role of the slowly excised U12 introns in down-regulation of gene expression.

**Reviewers:**

This article was reviewed by John Logsdon and Manyuan Long. For the full reviews, please go to the Reviewers' Reports section.

## Findings

Most of the eukaryotic protein-coding sequences are interrupted by multiple non-coding sequences, introns, which are excised from the transcript through the action of a complex molecular machine, the spliceosome, which consists of snRNP (small nuclear ribonucleoprotein particles) and hnRNPs (heterogeneous nuclear ribonucleoprotein particles), and is conserved throughout the eukaryotic world [1-3]. There are two types of introns, U2 and U12, which are excised by distinct spliceosomes, the major and the minor one, respectively; over 99% of the eukaryotic introns belong to the U2 class, and the remaining ones comprise the U12 class [4-6].

The atypical U12 introns have been recognized through their unusual splice junction structure, namely, |AT at the donor splice site and AC| at the acceptor splice site [7,8]. A closer examination of the sequences of these introns revealed additional features that distinguish them from the major U2 introns, including conservation of unusual signals at the donor splice site (|ATATCCTT) and in the vicinity of the acceptor splice site (TCCTTAAC 10–15 bases upstream of the splice junction). Subsequently, it has been shown that some |GT-AG| introns are also spliced out by the U12 spliceosome; as it turns out, actually, the majority of U12 introns are of the |GT-AG| type [9]. The U12 spliceosome was first identified and characterized in animals, where it has been shown to contain several unique small nuclear RNAs (snRNAs), including the eponymous U12, U11, U4atac, and U6atac, that are structurally similar and, apparently, functionally analogous to the snRNAs of the major spliceosome [10-12]. Recently, RNA and protein components of the minor spliceosome along with U12 introns have been also identified in plants, fungi, and unicellular eukaryotes [13,14]. Thus, the minor spliceosome and U12 introns that it removes have been detected in representatives of all eukaryotic supergroups for which substantial amounts of genome sequences are available; so it appears that the minor splicing system is as ancient as the major one [4,14].

Comparative analyses of the gene structures in orthologous genes from diverse eukaryotes have shown that up to 30% of U2 intron positions are conserved between animals and plants [15,16]. Combined with the demonstration that parallel gains of introns in the same position could account only for a relatively small fraction (~10%) of shared plant and animal intron positions [17,18], these findings indicate that a substantial fraction of introns in intron-rich extant genomes descends from the earliest stages of eukaryotic evolution.

The positions of U12 introns tend to be conserved among vertebrates [4,19], and two shared U12 intron positions have been detected in animal and plant genes for Na^+^/H^+ ^antiporters [20]. However, the overall level of conservation of U12 introns between plants and animals, and hence the depth of the evolutionary conservation of U12 introns is not known. We analyzed the available data on U12 introns in human and *Arabidopsis thaliana *genomes in order to systematically compare their conservation with that of U2 introns.

The U12 intron sequences were extracted from the SpliceRack database [21], and Arabidopsis and human genomic sequences were collected from NCBI. The U12 introns were mapped onto the genomic sequences yielding 570 human and 182 Arabidopsis U12 validated intron positions available for comparative analysis (Additional file 1). Probable orthologs were identified among human and Arabidopsis genes containing U12 introns by BLAST comparison, and the intron positions were mapped onto aligned protein sequences as previously described ([16] and Additional file 1). This procedure yielded 133 pairwise alignments of human-Arabidopsis orthologs with a total of 1796 intron positions (935 human and 861 Arabidopsis). Of these intron positions, 155 were conserved including 20 U12, 115 U2, and 20 "mixed" positions, with a U12 intron in one species and a U2 intron in the other. The fraction of shared intron positions was close to the previous estimates [16,18]. In agreement with the results previously reported for U2 introns [17], simulation of the intron distribution in the analyzed set of orthologous genes by random intron shuffling (10,000 simulations) among the identified intron positions showed that the probability to observe 20 U12 intron positions shared by human and Arabidopsis genes as a result of independent inrons gains is < 0.0001. Thus, the shared U12 introns, primarily, reflect *bona fide *evolutionary conservation. Moreover, and unexpectedly, the fraction of conserved U12 intron positions in the analyzed set of human/Arabidopsis orthologs was significantly greater than the fraction of conserved U2 introns (Table 1).

**Table 1:** Conservation of U12 and U2 intron positions in orthologous human and Arabidopsis genes^a^

	U2 intron positions		U12 intron positions		*P*_Fisher_
		
	Shared	Variable	Shared	Variable	
#conserved positions (U2–U12 mixed cases removed)	115	1371	20	115	0.008
#conserved positions, U2–U12 mixed cases are counted as variable introns	115	1391	20	135	0.003

^a^To eliminate potential artifacts caused by misalignment, all positions containing a deletion or insertion in the alignment within 5 adjacent position both upstream and downstream were discarded from calculation [16]; the results changed minimally when a stricter criterion was applied by eliminating 10 adjacent positions (data not shown).

Among the "mixed" positions, 15 contain U12 introns in the human genes opposite a U2 intron in Arabidopsis, and only 5 contains a U2 intron in the human gene opposite U12 in Arabidopsis. This significantly asymmetric distribution of the U12-U2 mixed sites (*P *= 0.02 according to the binomial test) is likely to reflect intensive U12 to U2 conversion in plant evolution which might be the reason behind the small number of U12 introns in Arabidopsis compared to humans.

**Figure 1:**
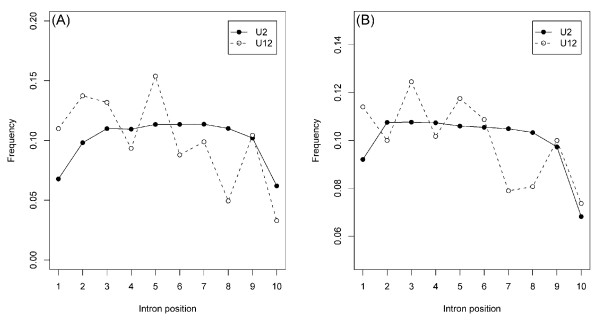
**Distribution of the positions of U12 and U2 introns across the length of the coding sequences in *Arabidopsis *(A) and human (B) genes**. For each intron, the distance from the 5' end of the coding sequence (after removal of intron sequences) was calculated and divided by the total length of the coding sequence. The resulting fractions were partitioned into 10 equal length bins. For comparing the distribution of intron in the 5' and 3' halves of genes, the total numbers of introns in bins 1–5 and in bins 6–10 were compared. The statistical significance of the difference between these numbers was determined using the two-sided binomial test, with the sum of these numbers treated as the total number of trials and the number of distribution in bins 1–5 as the number of successes, with prior probability of 0.5. **(A) **139,982 U2 and 182 U12 intron positions in *Arabidopsis *genes were analyzed. Among the U2 introns, 69,819 and 70,163 introns were contained in the 5' and 3'portions of genes, respectively (*P *= 0.3593). For U12 introns, 114 and 68 introns were contained in 5' and 3' portions of genes, respectively (*P*= 8.04 × 10^-4^). **(B) **230,339 U12 and 570 U12 intron positions in human genes were analyzed. Among the U2 introns, 119,949 and 110,390 introns were contained in the 5' and 3' region, respectively (*P*= 2.2 × 10^-16^). Among the U12 introns, 318 and 252 introns were present in the 5' and 3' region, respectively (*P*= 6.4 × 10^-3^).

It has been noticed previously that genes of intron-poor organisms display a substantial bias of intron distribution over the coding sequence length, with introns strongly over-represented in the 5'-portion of the genes, an observation that suggests a strong preference for intron loss in the 3'-portions of genes [22,23]. Moreover, even in intron-rich genomes, highly conserved, ancient introns concentrate in the 5'-portions of genes, suggesting the possibility of their preferential involvement in expression regulation and, possibly, other functional roles [24]. The distribution of U12 introns, in a sense, emulates the overall distribution of introns in intron-poor genome because there are so few representatives of this class of introns in any of the sequenced genomes. We, therefore, compared the distributions of U12 and U2 introns across the lengths of the coding sequences of human and Arabidopsis genes. As shown in Figure 1, in both organisms, the U12 introns show substantially greater enrichment in the 5'-portions of genes than the U2 introns. An even more notable observation was made when we compared the partitioning of conserved U12 intron positions and the mixed positions. There was a dramatic excess of conserved U12 intron positions in the 5'-portions of the analyzed genes and a reciprocal excess of apparent U12 to U2 conversions in the 3'-portions of Arabidopsis genes (Table 2).

**Table 2:** Distribution of conserved and mixed positions of U12 and U2 introns in human and Arabidopsis orthologs.

		Position		
Type	Number of introns	5'	3'	P-value^a^

Human U2/Arabidopsis U2	230	136	94	0.006734
Human U12/Arabidopsis U12	40	31	9	0.0006795
Human U12/Arabidopsis U2	30	9	21	0.04277
Human U2/Arabidopsis U12	10	6	4	0.7539

^a^Calculated using two-sided binomial test with prior probability 0.5 and the number of introns in the 5' half of a gene treated as the number of successes.

Although the number of U12 introns is small, calling for some caution in the interpretation of the results, taken together, these findings are compatible with the notion that U12 introns in 5'-portions of animal and plant genes tend to be conserved owing to their functional importance. It has been shown that the rate of removal of U12 introns from the respective transcripts by the minor spliceosome is several-fold slower than the rate of removal of U2 introns by the major spliceosome, leading to the hypothesis that U12 introns down-regulate the expression of their host genes [5,25]. The findings described here add credence to this hypothesis and suggest that recruitment of U12 introns for this regulatory role might account for their notable evolutionary conservation, and for the fact that U12 introns linger in numerous eukaryotic genome despite the ongoing, apparently, unidirectional conversion into U2.

## Competing interests

The authors declare that they have no competing interests.

## Authors' contributions

MKB performed sequence comparisons and contributed to the analysis of the results; WM contributed to the interpretation of the results; IBR contributed to the analysis of the results and wrote the initial draft of the manuscript; EVK incepted the study, contributed to the analysis of the results and wrote the final manuscript; all authors edited and approved the final version.

## Reviewers' comments

### Reviewer's report 1

#### John M. Logsdon, Jr., Department of Biology, University of Iowa, Iowa City, IO, USA

This brief contribution provides an interesting assessment of the conservation of spliceosomal introns comprising the major (U2) and minor (U12) classes. The analysis takes advantage of a recent comprehensive classification of introns into these two types (Sheth *et al*., 2006). Here, the authors focus on *Homo-Arabidopsis *comparisons; since the animal-plant split represents a deep divergence among eukaryotes, this should allow for inferences about early eukaryotic gene evolution. Previous comparisons of intron conservation between animal and plant genes have indicated that high fractions of intron positions are conserved. Since the fraction of U12 introns is less than 1% of all introns, these previous studies were necessarily focused on U2 introns (even though the introns were not explicitly classified as such). Since the types of introns can now be classified, the authors wished to explicitly compare levels and patterns of conservation among both U2 and U12 introns in *Homo *and *Arabidopsis *genes.

Of the 133 homologous *Homo *and *Arabidopsis *genes that contained at least one U12 intron, the authors compared conservation of intron positions. Of the 1796 positions, 155 were conserved, which is "close to the previous estimates" (although not 30%, the fraction suggested by some previous work). In any case, the key here is that there was a significant statistical excess of shared U12 introns, much higher than the fraction of shared U2 introns. Furthermore, of the "mixed" U2–U12 shared positions, a considerable majority are U12 type in *Arabidopsis*. Finally, the within-gene distribution of all U2 and U12 introns (not just the few shared ones), suggest a preference for U12 introns in 5' ends of genes; but when considering the shared U12 introns, the 5' bias is particularly prevalent in the shared U12 introns. The potential functional relationship between 5' introns and regulation makes this latter observation particularly interesting.

One weakness of this study is the fact that it relied solely on pairwise comparisons between two distantly related taxa (*Homo *and *Arabidopsis*). These and other authors have defended the hypothesis that the (high fraction of) similarly-identified animal-plant shared introns are mostly homologous, and that is a starting premise of this paper. However, such inferences remain untested by addition and consideration of many other intervening taxa (where the alternative hypothesis is that many shared introns have arisen by parallel insertion). Interestingly the shared U12 introns almost certainly represent homologous introns that can be traced to the animal-plant common ancestor; their rarity makes parallel insertion a highly improbable explanation. Perhaps this argument is similar for "mixed" introns, but the likelihood of parallel gain seems reasonably tenable. Overall, the paper is appropriate for publication in *Biology Direct*.

### Reviewer's report 2

#### Manyuan Long, Department of Ecology and Evolution, University of Chicago, Chicago, IL, USA

This manuscript reported a statistical analysis of the position conservation of two types of introns, U2 and U12. The U2 type has been found in 99% of eukaryotic introns. However, in the shared intron positions between plants and animals, this paper reports more conservation of U12 types, an unexpected and interesting asymmetric distribution of two types of introns. The interpretation for this observation is not a straightforward thing because of lack of outgroup to assign ancestral states. However, the authors proposed that this could be caused by the conversion of U12 to U2 introns in plant lineages. Their explanation is not unreasonable, because biologically the U12 splicing is not so efficient as U2 types so there could be a selective pressure against U2 to U12 conversion. Nevertheless, when more plant genomes are sequenced, the hypothesis that plant lineages are subject to higher rate of the U12 to U2 conversion may have opportunity to be tested by looking at the distribution of the turnover rates by comparing various branches of plants.

## Supplementary Material

Additional file 1 alignments of protein sequences of orthologous proteins from Arabidopsis and human employed for this analysis. Click here for file
